# Supplementary figures and images for: Differential scanning calorimetry of whole Escherichia coli treated with the antimicrobial peptide MSI-78 indicate a multi-hit mechanism with ribosomes as a novel target
Source: PeerJ. 2015 Dec 17;3:e1516. doi: 10.7717/peerj.1516 (PMC4690349; doi:10.7717/peerj.1516)

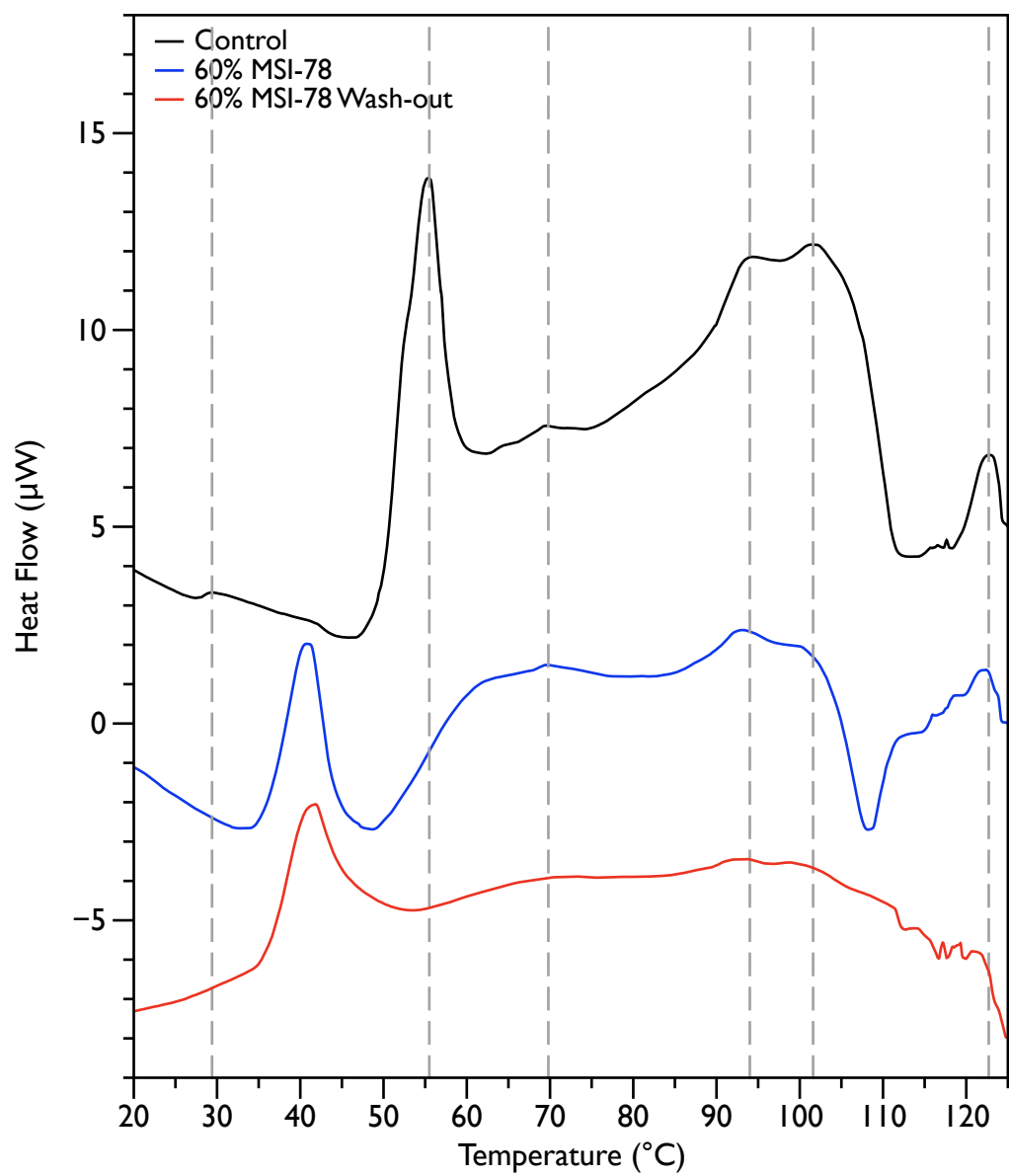

Supplement: Figure S1 [file peerj-03-1516-s001.pdf]
